# Quality and reliability of anxiety disorder-related short videos on TikTok and Bilibili: A cross-sectional study

**DOI:** 10.1097/MD.0000000000049431

**Published:** 2026-07-03

**Authors:** Biao Jiang, Qian-ying Liao, Xue-ping Yang, Yu-hao Chu

**Affiliations:** a Department of Neurology, Hanchuan People’s Hospital, Xiaogan, China; b Department of Neurology, The First Affiliated Hospital of Dali University, Dali, China; c Department of Neurology, Chaozhou People’s Hospital, Chaozhou, Guangdong Province, China.

**Keywords:** anxiety disorder, Bilibili, health communication, information quality, social media, TikTok

## Abstract

In recent years, short-form videos have demonstrated substantial potential in disseminating health-related information, with TikTok and Bilibili emerging as major platforms for public access to such content. However, to the best of our knowledge, no studies have systematically evaluated the quality and reliability of anxiety disorder-related videos on short-video platforms. Therefore, this study aimed to assess the quality and reliability of anxiety-related short videos on TikTok and Bilibili. A total of 250 anxiety disorder-related videos were analyzed from TikTok and Bilibili. Data on video characteristics, uploader identity, and engagement metrics were collected. The Global Quality Score and modified DISCERN were used to assess quality and reliability. The Mann–Whitney *U* test and Kruskal–Wallis *H* test were used for group comparisons, and Spearman rank correlation was applied to assess the relationships between engagement and video quality. The study revealed significant differences between the 2 platforms in video length, quality, reliability, and engagement metrics. Specifically, TikTok videos were shorter (median: 96 seconds, interquartile range [IQR]: 56.00–171.50) and received higher engagement (median likes: 5916, median shares: 2016) compared with Bilibili videos (median: 564 seconds, IQR: 200.00–1004.50, median likes: 611, median shares: 135). Videos uploaded by specialists exhibited higher quality and reliability, with a median Global Quality Score of 3.00 (IQR: 2.00–3.00) and a median modified DISCERN score of 2.00 (IQR: 2.00–3.00), significantly outperforming those uploaded by nonspecialists and individual users (*P* < .05). Content analysis indicated that most videos focused on symptoms (82.4%) and treatment (53.6%), while crucial topics such as diagnosis (9.2%), prevention (9.6%), and epidemiology (4.8%) were less frequently discussed. Anxiety disorder-related videos on TikTok and Bilibili have moderate quality and reliability. Specialist-uploaded videos are superior in quality. Key topics such as diagnosis and prevention should be better covered, and content moderation and professional involvement should be strengthened.

## 1. Introduction

Anxiety disorder is a common mental health issue that affects millions of people worldwide. Over 200 million people globally suffer from anxiety disorders, with the prevalence rising steadily in China, particularly among younger populations.^[1,2]^ Clinical manifestations of anxiety disorders include excessive worry, nervousness, palpitations, and insomnia, which can significantly impair daily functioning and quality of life.^[3]^ If left untreated, anxiety disorders can lead to more severe mental health issues, such as depression, worsened physical symptoms, and even increased suicide risk.^[4]^ Raising awareness about anxiety disorders is essential for early identification and timely treatment, alleviating both individual and societal healthcare burdens.

With the rapid growth of social media, platforms such as TikTok and Bilibili have become key channels for disseminating health information.^[5,6]^ Traditional texts and academic resources are often difficult for the general public to understand, whereas digital platforms have large user bases, personalized recommendation systems, and high interactivity, allowing medical information to spread quickly.^[7]^ These platforms convey concise health knowledge through short videos, engaging a large audience. However, concerns about the quality and reliability of these videos remain. Many studies show that video quality varies, with some spreading misleading or false information, potentially misguiding viewers about diseases and treatments and negatively impacting health behaviors.^[8–10]^ Despite the growing role of short-video platforms in health communication, the quality and reliability of anxiety disorder-related content have not been fully explored.

This study aims to assess the quality and reliability of anxiety disorder-related short videos on TikTok and Bilibili. By analyzing video content, engagement metrics, and uploader types, this study will provide insights into the quality characteristics of anxiety disorder-related videos on these platforms and suggest measures for improvement. The results are significant for enhancing the quality of health communication on digital platforms and promoting scientifically accurate public awareness of anxiety disorders.

## 2. Methods

### 2.1. Ethics approval

This study did not include human participants, clinical data, laboratory animals, or histological research. All data were collected from publicly available videos on TikTok and Bilibili in accordance with the respective platforms’ terms of service. Data collection was performed manually, and no automated scraping tools or application programming interfaces were used. Only publicly visible, aggregate information (e.g., video duration and engagement metrics) was recorded; no usernames, profile links, or other personally identifiable information were collected, and no interaction with platform users occurred. The study was non-interventional and observational in nature. Therefore, ethical approval was not required.

### 2.2. Search strategy and data extraction

**Figure 1.**
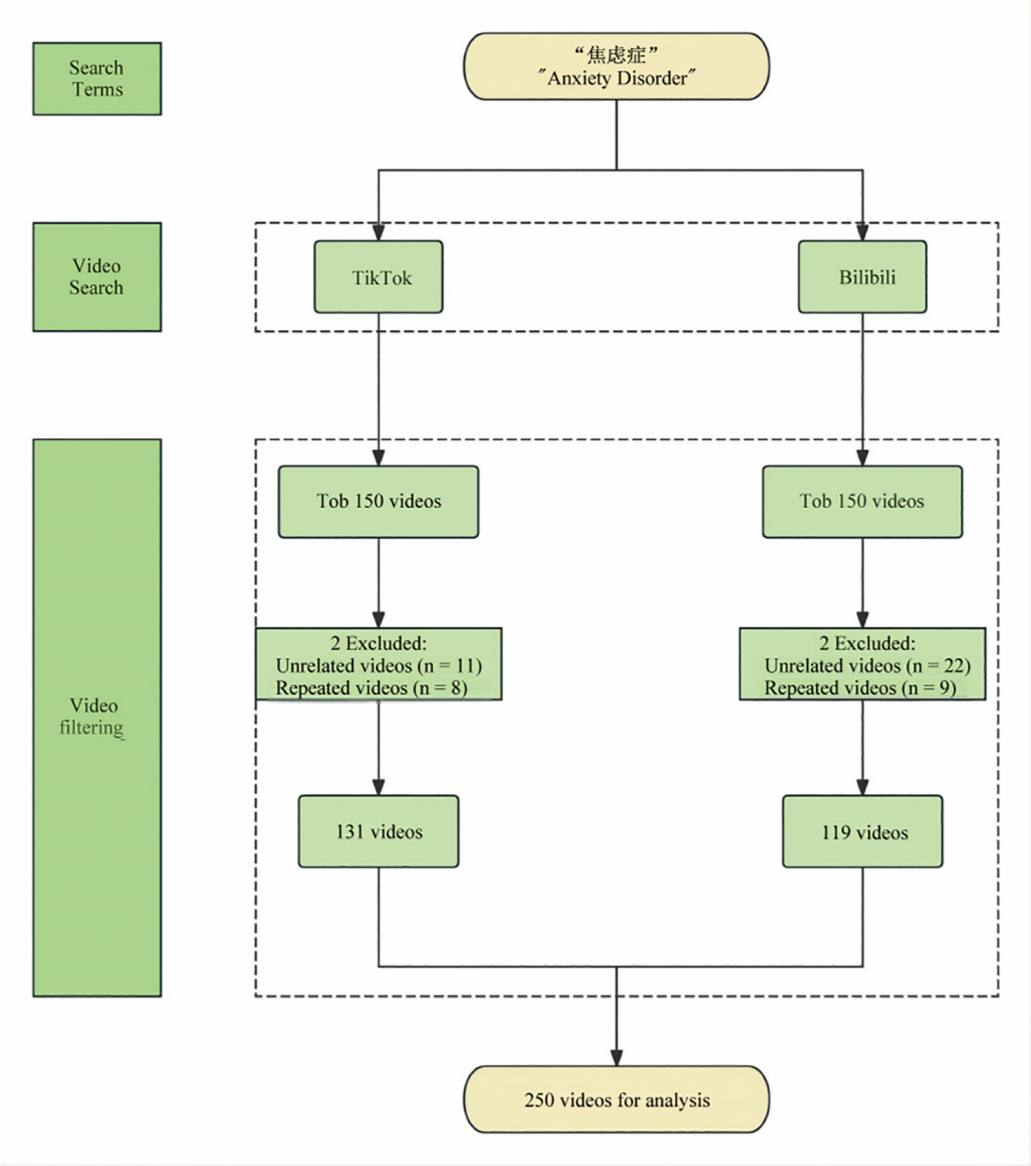
Flowchart of video selection.

This study employed a cross-sectional design, using the web-based platforms Bilibili (https://www.bilibili.com) and TikTok (https://www.douyin.com) as data sources. On September 27, 2025, a search was conducted using the Chinese keyword “焦虑症” (anxiety disorder). For each platform, the first 150 videos ranked by the default (comprehensive) sorting order were selected, and aggregate data on video duration, engagement metrics, and uploader identity were collected. Videos were collected without using any user accounts to minimize algorithmic bias, and all data were publicly available. To ensure language accessibility, only videos in Chinese or English with accurate Chinese subtitles were included. Exclusion criteria included videos unrelated to anxiety disorders, videos uploaded within the previous week, commercial advertisements or promotional content, and duplicate videos (the same content but different sources). For each eligible video, data were systematically extracted according to a predefined coding scheme, including video duration, engagement metrics (likes, comments, shares, and collections, defined as the number of times a video was saved or bookmarked), uploader type, Global Quality Score (GQS), modified DISCERN (mDISCERN) score, platform, and content, which was further categorized into 6 domains: epidemiology, etiology, clinical presentation, diagnosis, treatment, and prevention. In addition, videos were categorized according to uploader type as specialists, nonspecialists, and individual users. The study flowchart is shown in Figure 1, with the short-video data in the Supplementary Materials, Supplemental Digital Content 1.

### 2.3. Quality and reliability assessment

Between September 28 and 29, 2025, the quality and reliability of the videos were assessed using 2 validated tools: the GQS and the mDISCERN instrument. The GQS is a 5-point scale that evaluates video quality based on professionalism, clarity, and comprehensiveness of the content, where a score of 1 indicates poor quality and 5 represents excellent quality (Table 1).^[11]^ The mDISCERN is an adapted tool designed to evaluate the reliability of video information, with scores ranging from 1 to 5, where higher scores indicate greater reliability (Table 2).^[12]^ Each video was independently assessed by 2 trained raters with medical backgrounds (QL and XY), and any discrepancies were resolved through consultation with a third rater (YC) to reach consensus.

**Table 1:** The Global Quality Score quality criteria.

Item features	Points
Poor quality; poor flow of the videos; most information missing; not at all useful for patients	1
Generally poor quality; some information listed, but many important topics missing; of very limited use to patients	2
Moderate quality; suboptimal flow; some important adequately discussed, but other information poorly discussed; somewhat useful for patients	3
Good quality and generally good flow; most of the relevant information listed, but some topics not covered; useful for patients	4
Excellent quality and flow; very useful for patients	5

**Table 2:** The modified DISCERN quality criteria.

Reliability score
Is the video clear, concise, and understandable?
Are valid sources cited?
Is the content presented balanced and unbiased?
Are additional sources of content listed for patient reference?
Are areas of uncertainty mentioned?

### 2.4. Statistical analysis

Descriptive statistics were applied to summarize all variables. Continuous data were reported as mean ± standard deviation when normally distributed and as median with interquartile range (IQR) when non-normally distributed. Normality was assessed using the Shapiro–Wilk test and visual inspection of data distributions. Categorical variables were expressed as counts and percentages. Between-group comparisons of continuous variables were conducted using independent-sample *t* tests for normally distributed data, whereas the Mann–Whitney *U* test was used for nonparametric data. For comparisons involving 3 or more groups, the Kruskal–Wallis *H* test was applied, followed by the Dunn post hoc test for pairwise analysis where significant differences were detected. Bonferroni correction was applied to adjust for multiple testing in post hoc analyses. Correlations between video quality scores (GQS and mDISCERN) and engagement metrics (likes, comments, shares, and collections) were examined using Spearman rank correlation. Cohen kappa coefficients were used to evaluate inter-rater reliability for GQS and mDISCERN scores, with kappa values ≥0.8 indicating excellent agreement. Statistical significance was set at a two-tailed *P* value of <.05. All data analyses and figure generation were performed using R software (version 4.3.2).

## 3. Results

### 3.1. Video characteristics

This study analyzed 250 anxiety-related videos, showing significant differences in video length and engagement (Table 3). The median video length was 176.5 seconds (IQR: 83.25–590), with a median of 3079 likes (IQR: 734–9044), 1467 collections (IQR: 479–4241), 361.5 comments (IQR: 102.75–938.5), and 843 shares (IQR: 134.25–3353). Most videos focused on symptoms (82.4%), followed by treatment (53.6%) and etiology (28.4%). GQS and mDISCERN had median values of 3.00 and 2.00, indicating moderate quality.

**Table 3:** General characteristics, quality, and reliability of the videos.

Variables	Total (n = 250)
General information	
Video length (s), M (Q1, Q3)	176.50 (83.25, 590.00)
Likes, M (Q1, Q3)	3079.00 (734.00, 9044.00)
Collections, M (Q1, Q3)	1467.00 (479.00, 4241.25)
Comments, M (Q1, Q3)	361.50 (102.75, 938.50)
Shares, M (Q1, Q3)	843.00 (134.25, 3353.00)
Video content	
Epidemiology	12 (4.80%)
Etiology	71 (28.40%)
Symptoms	206 (82.40%)
Diagnosis	22 (8.80%)
Treatment	134 (53.60%)
Prevention	24 (9.60%)
Video quality	
GQS score, M (Q1, Q3)	3.00 (2.00, 3.00)
mDISCERN score, M (Q1, Q3)	2.00 (1.00, 2.00)

GQS = Global Quality Score, mDISCERN = modified DISCERN.

**Figure 2.**
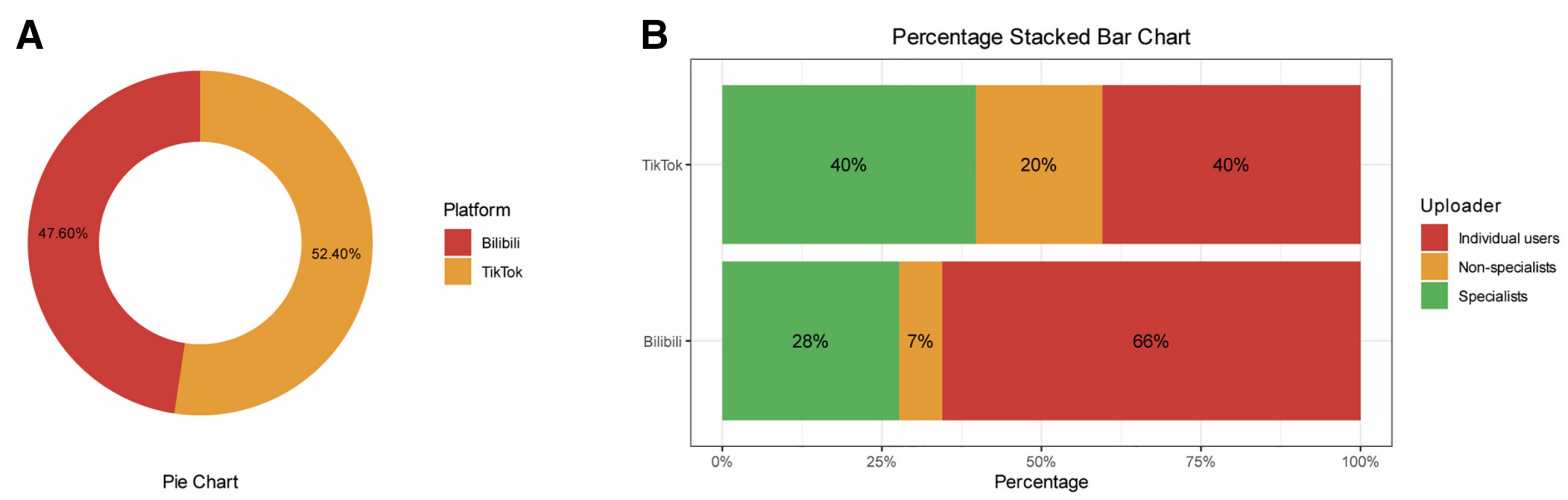
Distribution of video uploaders on Bilibili and TikTok. (A) Overall distribution of video uploaders. (B) Distribution of specialists, nonspecialists, and individual user uploaders on TikTok and Bilibili.

A total of 52.4% of the videos were uploaded to TikTok, whereas 47.6% were uploaded to Bilibili, as shown in Figure 2A. TikTok videos were shorter, with more likes, but Bilibili videos had slightly better GQS (Table 4). As shown in Figure 2B, on TikTok, 40.0% of the videos were uploaded by individual users, 20.0% by nonspecialists, and 40.0% by specialists. On Bilibili, 66.0% of the videos were uploaded by individual users, 7.0% by nonspecialists, and 28.0% by specialists. Specialists uploaded the longest videos with the highest GQS and mDISCERN scores. Individual users uploaded the longest videos, but their quality and reliability were significantly lower compared with specialists (Table 5).

**Table 4:** General information, quality, and reliability scores of anxiety disorder videos on TikTok and Bilibili.

Variables	Bilibili (n = 119)	TikTok (n = 131)	ES (95% CI)	*P*
General information				
Video length (s), M (Q1, Q3)	564.00 (200.00, 1004.50)	96.00 (56.00, 171.50)	−374.00 (−523.00, −279.00)	<.001
Likes, M (Q1, Q3)	611.00 (48.00, 2873.00)	5916.00 (3348.00, 13,359.50)	4381.00 (3580.00, 5668.00)	<.001
Collections, M (Q1, Q3)	728.00 (51.00, 2871.00)	2323.00 (1123.00, 4799.50)	1179.00 (794.00, 1712.00)	<.001
Comments, M (Q1, Q3)	105.00 (6.50, 318.00)	750.00 (360.50, 1440.50)	518.00 (390.00, 666.00)	<.001
Shares, M (Q1, Q3)	135.00 (3.00, 776.00)	2016.00 (845.50, 5093.00)	1505.00 (1116.00, 2027.00)	<.001
Video content				
Epidemiology	9 (7.56%)	3 (2.29%)		–
Etiology	34 (28.57%)	37 (28.24%)		–
Symptoms	92 (77.31%)	114 (87.02%)		–
Diagnosis	15 (12.61%)	7 (5.34%)		–
Treatment	72 (60.50%)	62 (47.33%)		–
Prevention	15 (12.61%)	9 (6.87%)		–
Video quality				
GQS score, M (Q1, Q3)	3.00 (2.00, 3.00)	3.00 (2.00, 3.00)	0.00 (0.00, 0.00)	.997
mDISCERN score, M (Q1, Q3)	2.00 (1.00, 3.00)	2.00 (1.00, 2.00)	0.00 (−1.00, 0.00)	.018

Between-group comparisons of continuous variables were performed using the Mann–Whitney *U* test.

Categorical variables were compared using the chi-square test.

All *P* values are two-tailed, and *P* < .05 was considered statistically significant.

95% CI = 95% confidence interval for effect sizes, ES = effect size, GQS = Global Quality Score, mDISCERN = modified DISCERN.

**Table 5:** Characteristics, quality, and reliability of anxiety disorder videos by different uploaders on TikTok and Bilibili.

Variables	Specialists (n = 85)	Nonspecialists (n = 34)	Individual users (n = 131)	ES	*P*	*P* *	*P* †	*P* ‡
Video length (s), M (Q1, Q3)	102.00 (58.00, 163.00)	90.00 (58.00, 121.25)	372.00 (176.50, 753.00)	0.203	<.001			
Likes, M (Q1, Q3)	3963.00 (857.00, 9522.00)	3702.50 (1774.50, 8489.25)	2873.00 (534.50, 9044.00)	0.001	.514			
Collections, M (Q1, Q3)	1466.00 (509.00, 3957.00)	1999.50 (1017.00, 4167.75)	1221.00 (283.00, 4515.00)	0.005	.335			
Comments, M (Q1, Q3)	406.00 (137.00, 853.00)	346.50 (133.25, 854.25)	350.00 (99.00, 1048.00)	−0.003	.828			
Shares, M (Q1, Q3)	1357.00 (123.00, 2979.00)	1619.50 (428.50, 2914.00)	665.00 (91.50, 3927.00)	0.016	.081			
GQS score, M (Q1, Q3)	3.00 (3.00, 3.00)	3.00 (2.25, 3.00)	2.00 (2.00, 3.00)	0.371	<.001	.177	<.001	<.001
mDISCERN score, M (Q1, Q3)	2.00 (2.00, 3.00)	2.00 (2.00, 2.00)	1.00 (1.00, 2.00)	0.227	<.001	.316	<.001	<.001

The overall *P* value was calculated using the Kruskal–Wallis *H* test.

When the overall test was significant, post hoc pairwise comparisons were performed using the Dunn test with Bonferroni correction.

ES = effect size, GQS = Global Quality Score, mDISCERN = modified DISCERN.

* *P* value for the comparison between specialists and nonspecialists.

† *P* value for the comparison between specialists and individual users.

‡ *P* value for the comparison between nonspecialists and individual users.

### 3.2. Video content

**Figure 3.**
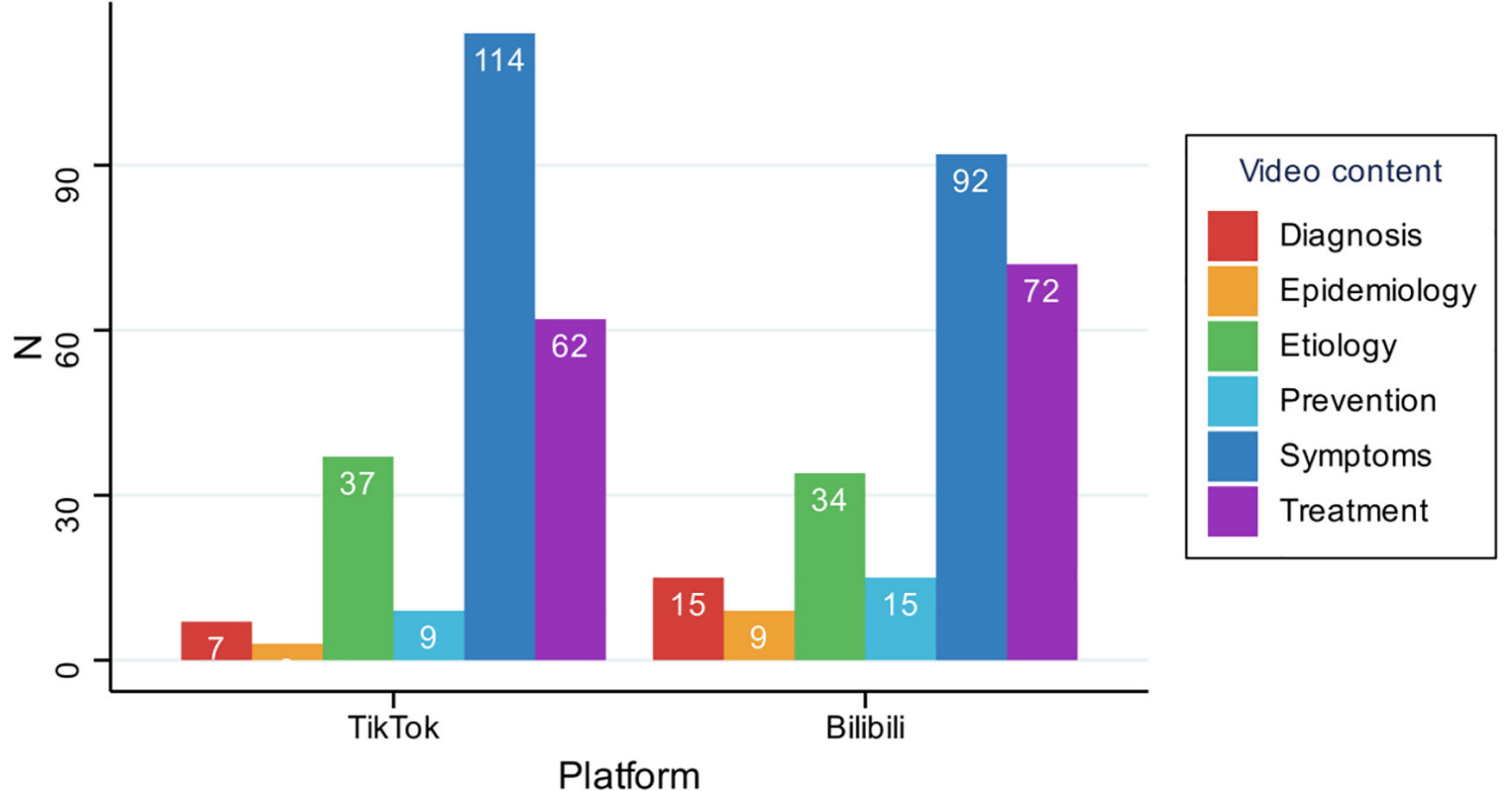
Information about anxiety disorder-related video content from TikTok and Bilibili.

Symptoms were the most common theme, appearing in 87.0% of TikTok videos and 77.3% of Bilibili videos (Table 4). Treatment was next (47.3% on TikTok, 60.5% on Bilibili). Etiology appeared in 28.6% of TikTok videos and 28.2% of Bilibili videos. Diagnosis was discussed in 12.6% of TikTok videos and 5.3% on Bilibili, while prevention was addressed in 6.9% of TikTok videos and 12.6% of Bilibili videos. Epidemiology was the least discussed, appearing in only 2.3% of TikTok videos and 7.6% of Bilibili videos, as illustrated in Figure 3.

### 3.3. Video quality and reliability

**Figure 4.**
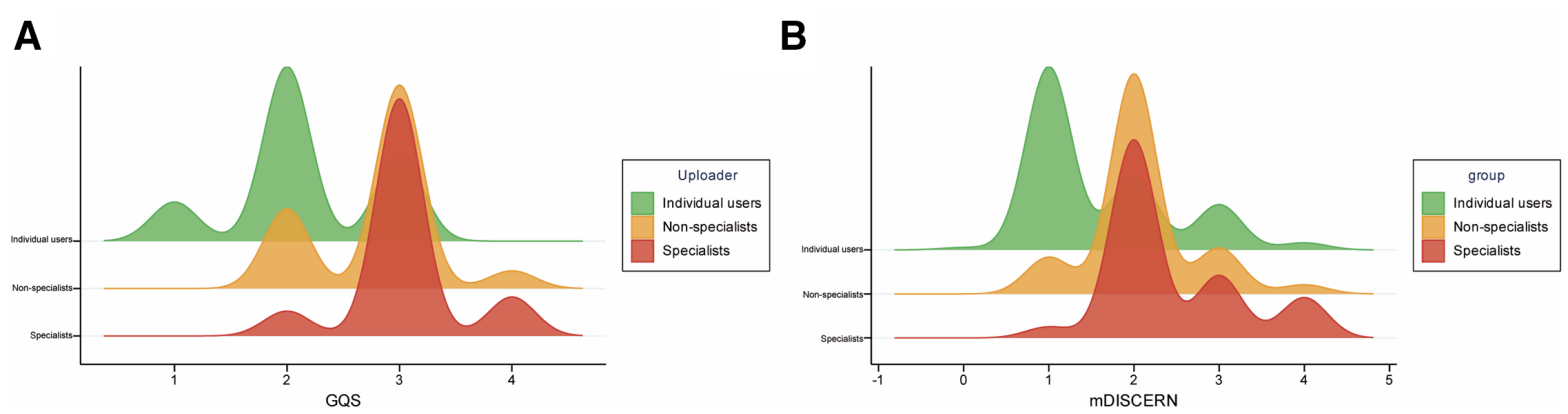
Distribution of video quality and reliability scores across uploader groups. (A) Distribution of GQS scores among uploader groups; (B) Distribution of mDISCERN scores among uploader groups. GQS = Global Quality Score, mDISCERN = modified DISCERN.

**Figure 5.**
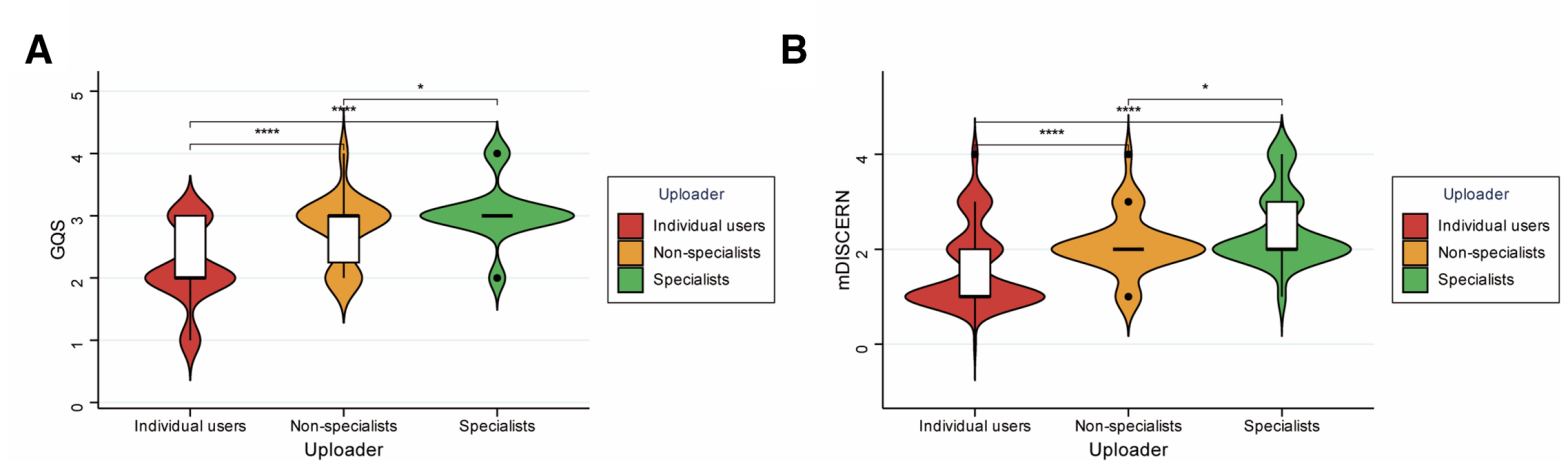
Comparison of quality and reliability scores between videos on TikTok and Bilibili. (A) Comparison of GQS scores among uploader groups; (B) Comparison of mDISCERN scores among uploader groups. GQS = Global Quality Score, mDISCERN = modified DISCERN.

The analysis revealed significant differences in GQS and mDISCERN scores between specialists, nonspecialists, and individual users (Table 5). Specialists consistently produced higher quality videos with a median GQS of 3.00 and a median mDISCERN score of 2.00. Nonspecialists and individual users had lower scores (Fig. 4A and B). Statistical significance was confirmed with *P* values below .05 for both GQS and mDISCERN scores (Fig. 5A and B).

### 3.4. Correlation between video features and quality

**Figure 6.**
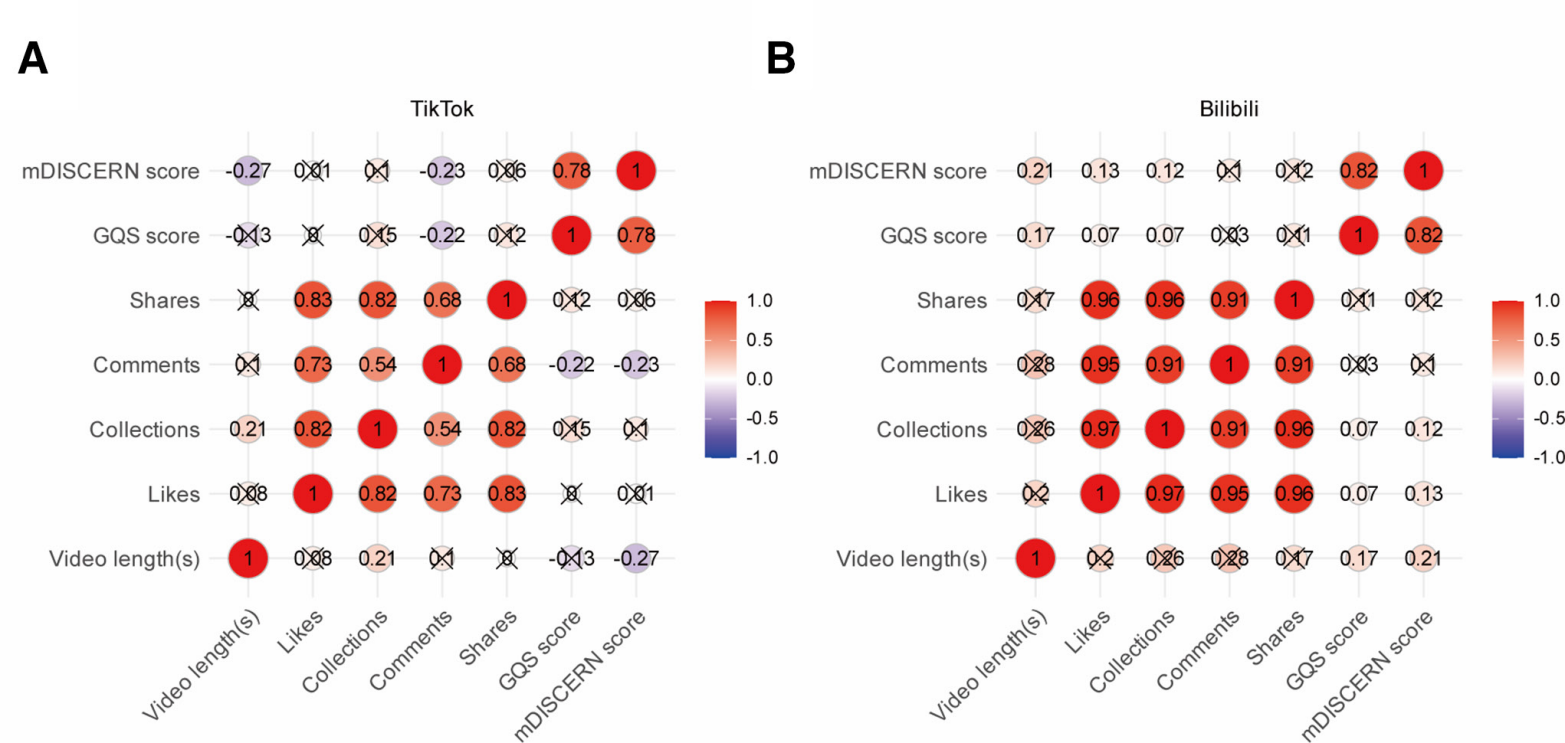
Correlation heatmap of video characteristics, engagement, and quality scores. (A) Correlation matrix for TikTok videos. (B) Correlation matrix for Bilibili videos. GQS = Global Quality Score, mDISCERN = modified DISCERN.

As shown in Figure 6A and B, significant relationships between GQS and mDISCERN scores were found on both platforms (*P* < .001). On TikTok, video length showed a negative correlation with mDISCERN (*r* = −0.27), and comments showed negative correlations with both GQS (*r* = −0.22) and mDISCERN (*r* = −0.23). On Bilibili, video length showed weak positive correlations with both GQS (*r* = 0.17) and mDISCERN (*r* = 0.21). Likes and collections exhibited weak positive correlations with GQS and mDISCERN, while shares had minimal correlations.

### 3.5. Video uploader

Videos were classified according to the uploader’s identity into 3 groups: specialists, including psychiatrists, clinical psychologists, and counselors; nonspecialists, including doctors from fields such as sleep medicine, pain medicine, traditional Chinese medicine, neurology, neurosurgery, general medicine, surgery, gynecology, dermatology, and cardiology; and individual users, including patients, for-profit organizations, nonprofit organizations, health promoters, and psychology students (Table 6).

**Table 6:** Uploader of short videos.

Uploader	Number (%)
Specialists	
Psychiatrists	41 (16.40)
Clinical psychologists	28 (11.20)
Counselors	16 (6.40)
Nonspecialists	
Sleep medicine	5 (2.00)
Pain medicine	3 (1.20)
Traditional Chinese medicine	6 (2.40)
Neurology	8 (3.20)
Neurosurgery	2 (0.80)
General medicine	4 (1.60)
Surgery	2 (0.80)
Gynecology	1 (0.40)
Dermatology	1 (0.40)
Cardiology	2 (0.80)
Individual users	
Patients	45 (18.00)
For-profit organizations	27 (10.80)
Nonprofit organizations	29 (11.60)
Health promoters	21 (8.40)
Psychology students	9 (3.60)

## 4. Discussion

This study evaluated the quality and reliability of 250 anxiety-related videos uploaded on 2 major Chinese social media platforms, TikTok and Bilibili. The analysis focused on video characteristics, engagement metrics, and differences across platforms and uploader types. The results revealed significant differences between the 2 platforms in terms of video length, engagement, and content quality. While short videos hold potential for effectively disseminating medical information, they also have notable limitations. Despite providing convenient and widespread access to health-related content, the overall quality and reliability of the videos were found to be lacking. Specifically, videos uploaded by specialists consistently outperformed those uploaded by nonspecialists and individual users, highlighting the critical role of professional involvement in ensuring the credibility and accuracy of health communication on digital platforms.

User engagement is commonly considered a surrogate indicator of influence in digital health communication.^[13]^ In this study, we observed that TikTok videos received significantly higher user engagement, with median values for likes, comments, collections, and shares all notably exceeding those on Bilibili. These results align with prior research, which suggests that TikTok’s short-video format, coupled with its algorithm-driven personalized recommendation system, promotes higher user interaction.^[14]^ In contrast, although Bilibili videos were generally longer, their engagement metrics were lower.^[15]^ This suggests that Bilibili’s user base may prioritize in-depth content consumption over rapid interactions.^[9,16,17]^ This observation is consistent with research indicating platform-specific differences in user behavior. TikTok’s entertainment-oriented design fosters quick and frequent interactions, attracting an active, predominantly younger audience. Meanwhile, Bilibili appeals to users who are more focused on educational content, tending to favor longer, more detailed videos.^[18,19]^ This implies that engagement patterns are influenced not only by platform algorithms but also by the distinct user behavior characteristics of each platform.

Regarding uploader types, our analysis revealed that videos uploaded by specialists generally exhibited better engagement metrics, despite being shorter in duration. This finding is consistent with the literature, which asserts that expert-led content is more likely to gain users’ trust, thereby enhancing engagement.^[10,20,21]^ In contrast, nonspecialists and individual users uploaded longer videos, but their interaction levels were lower. Many individual users, particularly patients with anxiety disorders, tend to express their experiences at length.^[3,22]^ However, due to the lack of professional expertise, their communication often lacks precision, leading to increased video duration without significantly improving engagement. This disparity underscores the critical role of professional knowledge in building user trust and engagement, which is vital for the effective dissemination of reliable health information.

The distribution of video content across the 2 platforms is highly uneven. Most videos focus on symptoms and treatment, while key topics such as epidemiology, etiology, diagnosis, and prevention are less frequently addressed. The absence of these topics raises serious concerns, as they are critical to enhancing public awareness, early diagnosis, and effective management of anxiety disorders. Epidemiology plays a crucial role in increasing awareness of the prevalence of anxiety disorders, helping reduce stigma and encouraging early intervention.^[23]^ The lack of explanation regarding etiology limits the public’s understanding of the disease’s mechanisms, which is essential for informed decision-making. If patients could understand the causes of anxiety disorders, such as neurotransmitter imbalances or amygdala dysfunction in the brain, they would have a clearer understanding of the disease and reduce the shame associated with it.^[24–26]^ Additionally, the lack of preventive content may hinder the promotion of lifestyle changes or coping strategies, which could reduce the incidence or severity of anxiety disorders.^[27]^ Without sufficient preventive information, patients may miss the opportunity to take management measures before the condition worsens.

The lack of focus on diagnosis is particularly concerning, as it could delay early detection, leading to inadequate treatment and disease progression. Many patients with anxiety disorders frequently visit various specialists due to somatic symptoms, such as palpitations or heart discomfort, but all diagnostic tests show no abnormalities. This not only wastes a significant amount of time and money but also increases distrust in the medical system.^[28,29]^ The failure to receive an accurate diagnosis may worsen anxiety symptoms and lead to additional health complications. The high comorbidity of anxiety disorders with physical diseases makes this issue more complex. Previous studies have shown that untreated anxiety disorders significantly increase the incidence of cardiovascular disease, hypertension, diabetes, and other conditions.^[30–32]^ Moreover, undiagnosed or untreated anxiety disorders can even lead to self-harm or suicidal tendencies in extreme cases.^[33]^ According to a Swedish nationwide study, psychiatrists recorded that nearly 30% of individuals with anxiety symptoms had suicidal thoughts, while only 7% of individuals without anxiety symptoms exhibited the same.^[34]^ Untreated anxiety disorders not only place a substantial burden on individual health but also have significant economic implications for society and the healthcare system. A previous study estimated that the annual economic burden of anxiety disorders in the United States is approximately 42.3 billion dollars.^[35]^ Additionally, a systematic review and meta-analysis published in 2024 found that the total economic cost of anxiety disorders exceeds 240 billion dollars.^[36]^ Missing opportunities for early intervention clearly constitute a serious public health issue, affecting both individual quality of life and leading to higher healthcare costs and longer recovery periods.

In this study, the overall quality and reliability of videos on TikTok and Bilibili were found to be at a moderate level, consistent with previous research. A study assessing breast cancer-related videos indicated that the quality and credibility of the videos were generally low, with the overall quality failing to meet satisfactory standards.^[6]^ Similarly, videos related to gastric cancer also exhibited deficiencies in both content and quality.^[37]^ These findings highlight that, while social media platforms can serve as powerful tools for disseminating health information, they often lack the rigorous review processes typical of traditional medical education, necessitating improvements in the quality and reliability of videos.

When comparing the platforms, no significant difference in GQS was observed between TikTok and Bilibili. The difference in mDISCERN scores was minimal, although Bilibili’s videos had a slightly higher range. This may be explained by TikTok’s shorter video format, which limits the comprehensiveness of content, and its algorithm-driven focus on entertainment, potentially diluting the educational value. In contrast, Bilibili’s longer videos allow for more detailed explanations.^[38]^ Bilibili users tend to view the platform as an educational resource, and many patients share their experiences and coping strategies, which could explain the higher quality of videos on Bilibili.^[39]^

This study examined the variations in quality and reliability across videos uploaded by specialists, nonspecialists, and individual users. The findings revealed significant discrepancies, with videos uploaded by specialists consistently outperforming those uploaded by nonspecialists and individual users in both GQS and mDISCERN scores. This pattern is in line with previous research in fields such as ophthalmology, cancer, and chronic disease management, which has consistently demonstrated that videos created by professionals are more accurate, evidence-based, and comprehensive than those created by nonexperts or individual users.^[10,17,20]^ However, the videos uploaded by nonspecialists and individual users exhibited lower quality and reliability, highlighting the critical need to ensure the accuracy of health information, especially for key conditions like anxiety disorders. It is worth noting that some individual user videos depicted anxiety disorders as “excessive worry” or “fragility,” which could contribute to misperceptions about the condition. Although specialist-uploaded videos showed significantly higher quality and reliability compared with other categories, the overall quality remained at a moderate level, as reflected in both GQS and mDISCERN scores. This indicates that there is still considerable room for improvement in content quality across all uploader categories. One possible explanation is that specialists, such as psychiatrists and psychologists, often prioritize professional content delivery through verbal explanations or dialogues with patients. While this ensures accuracy and reliability, it sometimes neglects more engaging or accessible presentation methods. For example, incorporating 3D animations that visually demonstrate neurotransmitter transmission or the manifestations of anxiety disorder episodes could enhance understanding and retention.^[40–42]^ This highlights the unique value of healthcare professionals in digital health communication and emphasizes the importance of making content both informative and accessible to a broad audience.

This study examined the relationship between engagement metrics (likes, comments, shares, collections), video duration, and video quality or reliability. Results showed weak correlations between engagement and video quality or reliability. Higher engagement did not significantly improve video quality or accuracy, and video length had a similarly weak correlation with quality. These findings suggest that engagement and video length alone are not sufficient indicators of content quality, underscoring the need for better content moderation.^[43,44]^

This study evaluated anxiety disorder-related videos on TikTok and Bilibili, analyzing video characteristics, engagement metrics, content focus, and uploader types. TikTok videos were shorter but had higher engagement than those on Bilibili. Expert-uploaded videos showed better quality, but key topics such as epidemiology, diagnosis, and prevention were underrepresented, limiting public awareness of anxiety disorders. Social media platforms can promote accurate health information by introducing verified clinician badges, embedding evidence-based reference links, and collaborating with clinicians and public health institutions to implement targeted content moderation. Health-related misinformation may encourage users to ignore professional medical advice, engage in self-diagnosis, adopt unverified treatments, and delay appropriate care, thereby worsening health outcomes. Moreover, misleading content can distort public understanding, hinder public health policy implementation, and exacerbate disease-related stigma. Growing evidence suggests that misinformation is increasingly prevalent in mental health-related online content, highlighting the need for strengthened digital health literacy interventions.^[45]^ Future research should focus on improving content moderation and encouraging expert-driven content.

Despite offering valuable insights, this study has several limitations. First, it focused solely on TikTok and Bilibili, excluding international platforms such as YouTube or Instagram. Including these platforms could offer a broader understanding of health video dissemination. Second, with a sample size of 250 videos, the findings may lack generalizability. Larger samples would enhance result robustness. Third, the evaluation process involved subjective elements. Although standardized tools were used, inter-rater differences may still exist. We also recognize that the GQS and mDISCERN, developed for long-form health content, may not be fully suitable for ultra-short videos. They may overestimate or underestimate video quality, as short videos often provide limited completeness, structure, and referencing.^[46]^ The absence of dedicated tools for health-related short videos suggests caution in interpreting results and highlights the need for specialized evaluation methods.^[47]^

## 5. Conclusion

This study found that anxiety-related videos on TikTok and Bilibili exhibited moderate quality and reliability. TikTok’s shorter videos had higher user engagement, whereas Bilibili’s longer videos offered more detailed content but received lower interaction. Videos uploaded by specialists demonstrated superior quality and reliability compared with those uploaded by nonspecialists and individual users. However, the focus of these videos was primarily on symptoms and treatment, with limited coverage of critical topics such as epidemiology, etiology, diagnosis, and prevention. Enhancing content in these areas, particularly through expert-led videos, is crucial for improving public understanding and effective management of anxiety disorders.

## Acknowledgments

The authors would like to express their gratitude to the participants who participated in the study.

## Author contributions

**Conceptualization:** Xue-ping Yang.

**Methodology:** Biao Jiang, Qian-ying Liao.

**Formal analysis:** Qian-ying Liao.

**Investigation:** Xue-ping Yang.

**Project administration:** Yu-hao Chu.

**Supervision:** Biao Jiang, Yu-hao Chu.

**Validation:** Biao Jiang.

**Writing – original draft:** Qian-ying Liao.

**Writing – review & editing:** Biao Jiang, Xue-ping Yang, Yu-hao Chu.


